# Imagistic Findings Using Artificial Intelligence in Vaccinated versus Unvaccinated SARS-CoV-2-Positive Patients Receiving In-Care Treatment at a Tertiary Lung Hospital

**DOI:** 10.3390/jcm12227115

**Published:** 2023-11-15

**Authors:** Alexandru Stoichita, Maria Ghita, Beatrice Mahler, Silviu Vlasceanu, Andreea Ghinet, Madalina Mosteanu, Andreea Cioacata, Andreea Udrea, Alina Marcu, George Daniel Mitra, Clara Mihaela Ionescu, Adriana Iliesiu

**Affiliations:** 1 Faculty of Medicine, University of Medicine and Pharmacy “Carol Davila”, 050474 Bucharest, Romania; beatrice.mahler@umfcd.ro (B.M.); vlasceanusilviu@yahoo.com (S.V.); adilies@yahoo.com (A.I.); 2 “Marius Nasta” Institute of Pneumology, 050159 Bucharest, Romania; andreea_bc15@yahoo.com (A.G.); madalina.mosteanu@yahoo.com (M.M.); andreea.cioacata@yahoo.com (A.C.); 3 Research Group of Dynamical Systems and Control, Ghent University, 9052 Ghent, Belgium; maria.ghita@ugent.be (M.G.); claramihaela.ionescu@ugent.be (C.M.I.); 4 Faculty of Medicine and Health Sciences, Antwerp University, 2610 Wilrijk, Belgium; 5 Faculty of Medicine, University of Medicine and Pharmacy of Craiova, 200349 Craiova, Romania; 6 Medicai, 020961 Bucharest, Romania; andreea@medicai.org (A.U.); alina@medicai.org (A.M.); george@medicai.org (G.D.M.); 7 Automation Department, Technical University of Cluj-Napoca, 400114 Cluj-Napoca, Romania; 8 Clinical Hospital “Prof. Dr. Th. Burghele”, 061344 Bucharest, Romania

**Keywords:** lung lesion, SARS-CoV-2, vaccination, image interpretation, artificial intelligence

## Abstract

Background: In December 2019 the World Health Organization announced that the widespread severe acute respiratory syndrome coronavirus 2 (SARS-CoV-2) infection had become a global pandemic. The most affected organ by the novel virus is the lung, and imaging exploration of the thorax using computer tomography (CT) scanning and X-ray has had an important impact. Materials and Methods: We assessed the prevalence of lung lesions in vaccinated versus unvaccinated SARS-CoV-2 patients using an artificial intelligence (AI) platform provided by Medicai. The software analyzes the CT scans, performing the lung and lesion segmentation using a variant of the U-net convolutional network. Results: We conducted a cohort study at a tertiary lung hospital in which we included 186 patients: 107 (57.52%) male and 59 (42.47%) females, of which 157 (84.40%) were not vaccinated for SARS-CoV-2. Over five times more unvaccinated patients than vaccinated ones are admitted to the hospital and require imaging investigations. More than twice as many unvaccinated patients have more than 75% of the lungs affected. Patients in the age group 30–39 have had the most lung lesions at almost 69% of both lungs affected. Compared to vaccinated patients with comorbidities, unvaccinated patients with comorbidities had developed increased lung lesions by 5%. Conclusion: The study revealed a higher percentage of lung lesions among unvaccinated SARS-CoV-2-positive patients admitted to The National Institute of Pulmonology “Marius Nasta” in Bucharest, Romania, underlining the importance of vaccination and also the usefulness of artificial intelligence in CT interpretation.

## 1. Introduction

Over the last decades, coronaviruses have been associated with significant disease outbreaks in East Asia and the Middle East, more specifically severe acute respiratory syndrome (SARS) emerged in 2002 and Middle East respiratory syndrome (MERS) emerged in 2012. The last outbreak is the most extended one and started in late 2019, with its first case in Wuhan City, Hubei Province, China, on 12 December 2019, causing an ongoing pandemic in most countries worldwide [1].

Coronaviruses (CoVs) are a subfamily of the family Coronaviridae called Orthocoronavirinae. Positive-sense, single-strand RNA (+ssRNA), measuring 27–32 kb, is the second biggest of all RNA virus genomes. CoVs contain an encased, crown-like viral particle [2]. Like other RNA viruses, SARS-CoV-2 is highly susceptible to genetic evolution while adapting to new hosts. Since the pandemic started, several variants have been described, but only a few had a great impact on global public health due to their virulence. Some of these variants, such as ALPHA (first discovered in the UK, November 2020), BETA (South Africa, October 2020), GAMMA (Brazil, December 2020), DELTA (India, December 2020), and the last highly spread one, OMICRON, which was discovered in December 2021 in South Africa, are considered variants of concern (VoC) while some others are considered variants of interest (VoI) [3]. 

Patients with COVID-19 exhibit a variety of symptoms, including fever, cough, exhaustion, dyspnea, sputum production, and headache associated with bilateral pulmonary infiltrations [4]. In vulnerable patients, viral pneumonia can rapidly become severe metabolic acidosis, acute respiratory distress syndrome (ARDS), as well as sepsis and septic shock [5]. 

The COVID-19 severity spectrum varies from mild or moderate to severe or critical illness that can be fatal. The World Health Organization (WHO) considers the relationship between oxygen dependency, disease severity, and mortality when classifying individual patients clinically [6]. With over 400 million cases and over 5 million deaths worldwide, every country has been impacted by the effects of SARS-CoV-2. In Romania, there have been over 2.5 million infections reported including deaths, since the first official case which was detected in February 2020 and over 8 million people have been vaccinated with the complete scheme [7].

Currently, the only effective method in preventing severe forms of SARS-CoV-2 is vaccination. Different types of vaccines are approved worldwide such as mRNA vaccines and viral vector vaccines [8]. In Romania, over 8 million people have been vaccinated with the complete scheme, representing 49% of the population, which is the second-lowest vaccination uptake of EU countries [7,9].

The CT scan is one of the most crucial management tools for hospitalized SARS-CoV-2-positive patients since lung damage is the major cause of mortality. The CT scan can assist the physician in diagnosing, detecting problems, and treating patients [2,10]. Furthermore, new advancements in artificial intelligence (AI) have supported the medical staff in their day-to-day practice. Without a doubt, the most talked-about topic in current medical research, both for diagnostic and therapeutic purposes, is AI or machine learning, as a helpful tool in the decision-making process of the clinician. 

AI has been used by researchers to automatically detect intricate patterns in imaging data and to provide quantitative evaluations of radiographic traits [11]. In radiology, AI is effectively used to detect specific characteristics even if they are not perceptible to the human eye. Perceived as a perceptual science, radiology is currently becoming more objective [12,13]. AI has been successfully used in radiation oncology for automatic tumor and organ segmentation as well as tumor monitoring for adaptive treatment [14,15,16].

Research is limited regarding the imagistic findings among SARS-CoV-2 vaccinated versus unvaccinated patients in Romania. In this study, we addressed this knowledge gap. The aim of this study is to investigate the presence of pulmonary lesions in vaccinated versus unvaccinated COVID-19 patients receiving in-patient care at the National Institute of Pneumonology (NIP) “Marius Nasta” in Romania from September 2021 to January 2022. AI software was used to automatically evaluate the pulmonary damage caused by SARS-CoV-2 from the thoracic CT scans of the patients. Three algorithms were implemented: production, research I, and research II. The specific objectives of the study were to describe the socio-demographic characteristics of the studied population, to assess the percentage of lung lesions from imagistic tools in the vaccinated population versus the unvaccinated population, to compare the evaluation obtained through AI tools with the medical evaluation provided by the clinician, and to provide statistical analysis and discussion on the results.

## 2. Materials and Methods

### 2.1. Clinical Study Design and Population

The clinical study was a monocentric, retrospective, cohort study using primary data collection. The study received ethical approval from the Ethics Committee of NIP “Marius Nasta”. The protocol has been created in accordance with all criteria and guidelines for clinical observational studies. Because the study site complies with GDPR regulations, all data collected for the study will be held in the strictest confidence. All personal data have been anonymized.

Inclusion criteria: The study included 186, adults (≥18 years old) diagnosed with COVID-19 and receiving in-patient care at the National Institute of Pneumonology “Marius Nasta” in Romania from September 2021 to January 2022. All included patients were admitted to the hospital with mild/moderate to severe symptoms associated with pulmonary lesions on chest X-rays. The patients were assessed using CT scans within the first week of hospitalization. Each patient signed an informed consent form (ICF) authorizing his/her participation in the research and inclusion in the study.

Biometric information of the included patient population such as age, sex, and comorbidities was collected from each patient’s medical record, along with data regarding the vaccination/non-vaccination for SARS-CoV-2 infection, vaccine type and the number of doses, and imaging data (thorax CT and X-ray scans). The bilateral pulmonary lesions evaluated by the pulmonologist were provided for 14 patients, assigning a percentage range to describe the impairment caused to the lungs. Due to data availability, the group of 14 subjects was chosen for training the algorithms of the AI software in order to determine the percentage of pulmonary lesions in 124 patients from the database with lung lesions and accessible CT scans. The study aimed to provide an interpretation of the results for these participants carried out with AI tools.

### 2.2. Data Processing

The CT scans of SARS-CoV-2 infected patients were analyzed by artificial intelligence software provided by Medicai Infrastructure (Medical Imaging Infrastructure Solutions, Bucharest, Romania, Pyton code for software, V1). The software allows the real-time integration of specialized AI algorithms, developed at a centralized level in Medicai infrastructure, complying with GDPR and HIPAA regulations. Medicai is a cloud-based healthcare tool used for digitizing medical data and imaging, employing AI solutions that are implemented directly in the platform.

The interpretation of medical images from CT scans requires the segmentation of lungs and corresponding lesions performed layer by layer. The architecture used for medical imaging segmentation in the current experiment is a deep convolutional network first proposed by Ronneberger et al. [1] named U-Net. These U-Net network and training strategies used to identify each lung, as well as the lung lesions due to COVID-19 disease are preferred due to the following advantages that overcome the challenges encountered during image segmentation: U-shaped structure, fast training speed with low training data availability, and identification of overlapping objects, fuzzy borders, and objects with shape variations and low contrast edges. Since U-Net is based on a fully convolutional network (FCN), a comprehensive methodology is provided in [2]. In a recent article, Yin et al., review and categorize the medical image segmentation technologies based on the U-Net structure and related deep learning network methods [3].

**Figure 1:**
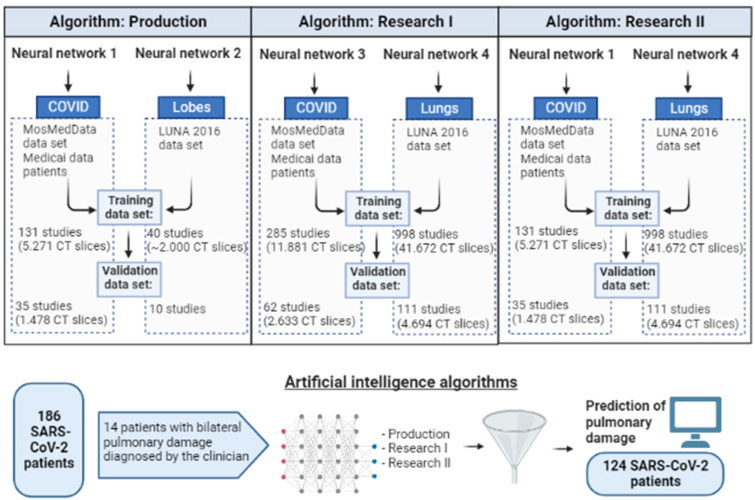
Representation of the algorithms used by the artificial intelligence software: production, research I, and research II; event schedule for the study methodology.

The automatic interpretation of pulmonary damage in thoracic CT scans was accomplished by implementing 3 algorithms: production, research I, and research II. All algorithms have been trained and validated using data containing COVID-19 characteristics and nodules previously detected. Figure 1 presents an overview of the algorithms and the flow diagram of the events schedule. The performed algorithms use publicly available CT datasets [7,8] and CT datasets from the patients’ files of this study archived in the Medicai platform. The published large-scale datasets contain anonymized human CT scans with features for COVID-19 patients (MosMedData [7]) and pulmonary nodules in lung cancer patients (LUNA 2016 [8]).

### 2.3. Data Analysis and Statistics

The data analysis was conducted using IBM^®^ SPSS^®^ Statistics SPSS (V22). Data entry errors were identified and cleaned through random spot-checking. The study variables were summarized using frequencies for categorical variables and respective descriptive statistics for continuous variables (means with standard deviations or medians with interquartile ranges). 

Statistical comparisons were performed using Student’s *t*-test, one-way ANOVA, and boxplots, with *p* values < 0.05 considered statistically significant. Each boxplot indicates the central mark as being the median, and the bottom and top edges of the box indicate the 25th and 75th percentiles, respectively. 

## 3. Results

### 3.1. Characteristics of the Study Population

The study included a cohort group of 186 SARS-CoV-2-positive patients investigated at the National Institute of Pulmonology “Marius Nasta” in Bucharest, Romania, with 79 (42.47%) females and 107 (57.53%) males. To compare the differences among the patients that can influence their health status, the socio-demographic characteristics of the studied population are analyzed and depicted in Table 1. The majority of the study participants were above 50 years old, with 83% incidence, while 84% of them were not vaccinated. From these data, we can see that over five times more unvaccinated patients than vaccinated ones are admitted to the hospital and require imaging investigations. Following the fact that 147 (79%) of the patients have concomitant comorbidities, significant evidence shows the possible impairment of the lungs provoked by SARS-CoV-2 in these patients.

**Figure 2:**
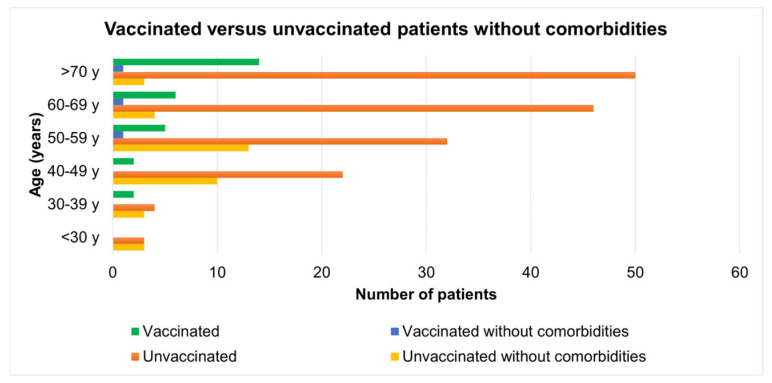
Incidence of vaccinated and unvaccinated patients without comorbidities grouped by age category.

A comparison of vaccinated versus unvaccinated patients without comorbidities reveals that 10.34% of vaccinated persons required imaging recommendations and hospitalization, while the number is doubled for unvaccinated patients (22.92%). Even without having any comorbidities, older patients tend to be more predisposed to need an additional imaging test during hospital visits. The comparison of the number of patients depending on specific categories is depicted in Figure 2.

Out of 186 patients, a total of 147 (79%) patients were identified as having comorbidities. As shown in Table 2, the most common comorbidities were cardiovascular: hypertension (25.85%), diabetes (18.37%), chronic myocardial ischemia (11.56%), ischemic stroke (6.12%), and atrial fibrillation (3.40%). Other pulmonary diseases were prevalent in the study (Table 2).

### 3.2. Training AI Algorithms to Interpret Imagistic Lung Lesions

In this study, we explored the accuracy of pulmonologists’ diagnosis of bilateral pulmonary damage and the variability of results when interpreting CT scans compared with artificial intelligence-based algorithms. The AI software examined the cases of 14 patients who were diagnosed by the medical team and received a percentage range characterizing their bilateral pulmonary damage. The pulmonologists assigned a preferred range based on personal expertise and complete clinical information acquired with PFTs and CT imaging. These data were obsolete for training the three algorithms used in this study for further interpretation of CT images of the remaining patients without a medical quantification of the lung damage.

**Figure 3:**
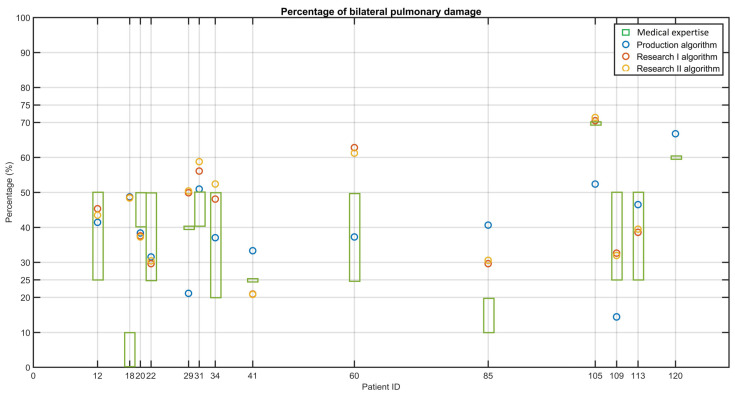
Percentage of bilateral damage per patient given by: medical expertise (green intervals), the production algorithm (blue circles), the research I algorithm (red circles), and the research II algorithm (yellow circles). The AI-based interpretation matches the interpretation of the physician when the rectangle matches the circles.

Figure 3 shows the results obtained from AI software using the production algorithm (blue circles), research I algorithm (red circles), and research II algorithm (yellow circles). The percentage of bilateral pulmonary damage given by the doctor is represented as green squares for each patient ID. When compared with the clinical diagnosis, the AI software assigned a correct percentage in 64.28% of the cases with production and research I algorithms, proving that it may serve as a decision-support tool for clinical practice. For the research II algorithm, 57.14% of cases were correctly estimated. 

An advantage of using computer algorithms is reducing the amount of time for patient data interpretation; using the algorithms it is 5 min for one patient, in comparison with a medical consultation of 30 min.

The results presented used the Bone Nativ 2.5 mm series from the CT database, giving the most significant performance. Hence, the rest of the patients were analyzed using the AI algorithms for assigning a correct percentage category for lung bilateral damage, without having the medical interpretation.

### 3.3. Characterization of Imagistic Lung Lesions Using AI Algorithms

**Figure 4:**
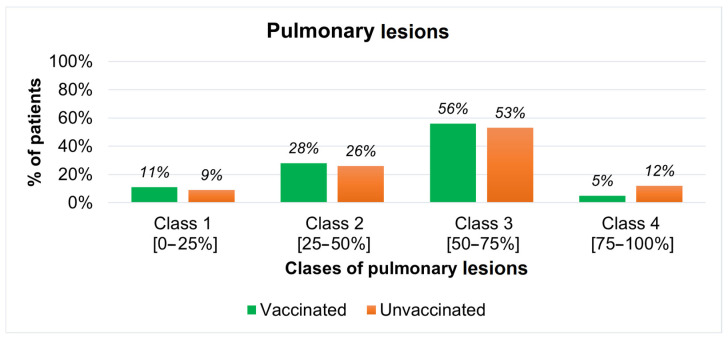
Patients’ incidence by classes of pulmonary lesions.

Out of 186 patients, only 124 (66%) had a CT imaging investigation with the artificial intelligence component. The patients were divided into four classes of lung damage, which can be seen in Figure 4, showing the highest percentage of patients having between 50 and 75% pulmonary damage. Furthermore, more than double unvaccinated patients compared to vaccinated ones have been found to have more than 75% of their lungs affected. This is a remarkable outcome since the number of unvaccinated patients is 5 times higher than the ones that received a SARS-CoV-2 vaccine with different numbers of shots.

Patients in the age group 30–39 have had the most lung lesions at almost 69% of both lungs affected. After comparing the results obtained by the AI and the results from the medical professionals, the software’s accuracy was 86%.

**Figure 5:**
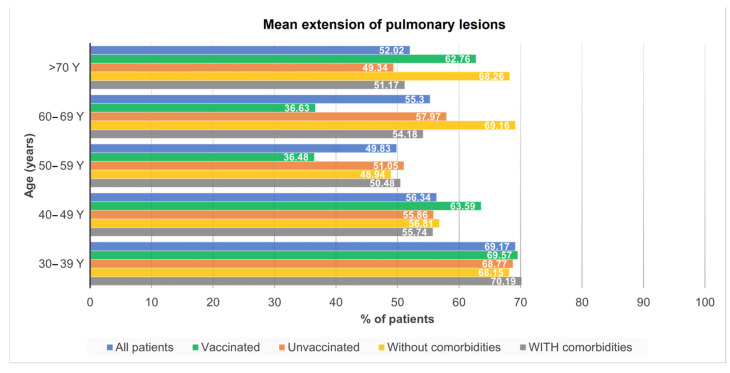
Mean extension of pulmonary lesions by age groups, in different categories of patients: all patients, vaccinated patients, unvaccinated patients, patients without comorbidities, and patients with comorbidities.

The mean extension of pulmonary lesions was assessed and illustrated in Figure 5. Patients in the age group 30–39 have had the most lung lesions with almost 70% of both lungs affected. Both patients with and without comorbidities showed a prevalence of pulmonary lesions higher than 50%. In contrast, the mean extension of pulmonary lesions in vaccinated subjects was significantly lower for the group of patients aged 50–69 years.

**Figure 6:**
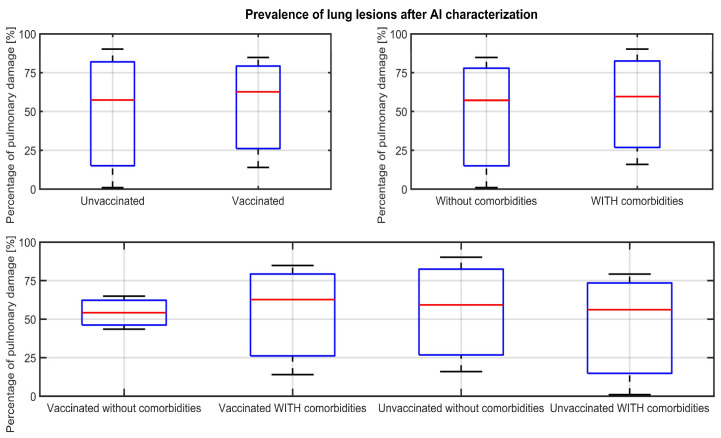
Boxplots for comparison of lung lesion prevalence per each patient category.

Applying the AI algorithms to the 124 admitted cases of patients with CT revealed that the population consisted of a mean of 53.83% lung lesions in unvaccinated patients, 54.39% in vaccinated patients, 57.14% in patients without comorbidities, and 52.92% in patients with comorbidities. Compared to unvaccinated patients without comorbidities, the vaccinated ones have a lower mean extension of lung lesions (54.18%). The boxplots in Figure 6 display the distribution of lung lesions for each corresponding category of patients. There were no statistically significant differences in the extension of lung lesions between vaccinated versus unvaccinated patients (*t*-test, *p* > 0.005).

Comparing the results obtained by the AI with the results from the medical professionals, the software’s accuracy was 86%.

## 4. Discussion

In this study, we performed the assessment of lung lesion quantification in SARS-CoV-2-positive patients using AI software to interpret the CT scans of the patients. The aim of the study was to characterize the population studied and to provide a quantification of the lung lesions of SARS-CoV-2 hospitalized patients, in order to allow the medical community to objectively assess the lung condition of the patient, as a complementary tool in the clinical decision-making process. The previous evaluation of lung function in individuals who have experienced SARS-CoV-2 infection has unveiled a spectrum of lung damage severity, shedding light on the intricate and multifaceted consequences of the virus on respiratory health [17,18]. We demonstrated that the designated combination of AI algorithms had an accuracy of 86% compared to the individual score provided by the medical professionals for the prevalence of lung lesions in SARS-CoV-2 patients.

Studies have validated and shown the valuable role of AI versus clinicians’ assessment in medical imaging for digital pathology applications [9]. The main challenges are imposed by the large-scale multi-dimensional dataset and the reliability of the interrogation of data characteristics across engineers and clinicians [10]. Deep learning methods are currently used for the automatic identification of physiological structures, dominating the in-depth characterization of tissue pathophysiology [19]. The adoption of current algorithms developed by Medicai is motivated by the software’s accuracy in image classification, segmentation, and performance [20].

Recently, AI-based applications and other mathematical techniques have been employed in research opportunities for different applications regarding lung characterization in several pathologies [21,22,23]: the novel severe acute respiratory syndrome-coronavirus-2 pandemic, asthma, and COPD; interpretation of pulmonary function tests, thoracic CT screening, patient’s prognosis in lung cancer, and others in the practice of pulmonology medicine [24,25,26,27,28,29,30,31]. According to the current evidence, AI, machine learning, and engineering tools are mainly used in the management of respiratory diseases, complementary to human experts’ decisions.

Furthermore, an important advantage of using computer algorithms was the shortened time for the patient data evaluation. In our study, the time for CT scan analysis using AI was reduced to 5 min for one patient compared with a medical consultation duration of 30 min. This underlines the utility of AI when a high number of patients need to be assessed in a short period of time, such as during pandemics.

In an attempt to transfer learning approaches, emerging multi-level patient information and imaging data jointly with medical interpretation of patients’ diagnosis elevates the quality-of-care level and clinical outcome for the patient in the hospital. For that purpose, clinical investigators need considerable research to begin the adoption of the presented methods in clinical practice.

Therefore, the study has several limitations. The study population is highly represented by unvaccinated patients, which unfortunately is representative of the general population in Romania. This limitation was beyond our control, as it reflects the real-world scenario during the study period. Furthermore, the study was conducted with the data available to us at the time, and we faced limitations in obtaining additional data, especially in terms of different vaccine regimens. We have taken care to present the data accurately and transparently within the constraints of our available resources, emphasizing that vaccines can fortify the defense against infectious diseases [32]. The AI software is limited in diagnosis since several additional factors that are not included in the software features are usually quantified by clinicians (e.g., patient history, concomitant respiratory diseases, specific symptoms, additional pulmonary function test results, and clinical information). We can speculate that the AI interpretation favors the medical decision in this study cohort. Also, the degree of lung damage can be influenced by the time when the CT scans were assessed, with the variability between patients’ scanning time during disease evolution being important. Moreover, future research should incorporate a larger dataset for training and validating the algorithms, since the test sample of 14 patients may not entirely reflect the prevalence of lung damage that clinicians confront in daily practice in post-SARS-CoV-2 patients.

## 5. Conclusions

In hospitalized unvaccinated SARS-CoV-2-positive patients, the extent of pulmonary lesions was higher than in vaccinated patients, confirming the protective role of vaccination. The role of artificial intelligence, by reducing the time for analyzing the CT imaging data of the patients, turns out to be an important additional diagnostic tool supporting clinicians in disease management by rapidly assessing large numbers of patients, particularly during pandemics.

## Figures and Tables

**Table 1:** Characteristics of included patient population (*n* = 186).

Characteristic	Value (*n* = 186 Patients)	% of the Population
Sex		
Female	79	42.27%
Male	107	57.53%
Age (years)		
<30	3	1.61%
30–39	6	3.23%
40–49	24	12.90%
50–59	37	19.89%
60–69	52	27.96%
>70	64	34.41%
Comorbidities		
Yes	147	79.03%
No	39	20.97%
SARS-CoV-2 vaccination		
Vaccinated	29	15.59%
Pfizer (2 shots)	17	9.13%
Pfizer (1 shot)	1	0.53%
Pfizer (unspecified number of shots)	1	0.53%
Astra Zeneca (2 shots)	5	2.68%
Moderna (2 shots)	1	0.53%
J&J/Janssen	4	2.15%
Non-vaccinated	157	84.40%

**Table 2:** Comorbidities of the included patient population (*n* = 147).

Comorbidity	Value (*n* = 147 Patients)	% of the Population
Hypertension	38	25.85%
Diabetes	27	18.37%
Chronic Myocardial Ischemia	17	11.56%
Ischemic Stroke	9	6.12%
Asthma	6	4.08%
Hyperlipidemia	5	3.40%
Chronic Renal Disease	5	3.40%
Atrial Fibrillation	5	3.40%
Myocardial Infarction	4	2.72%
Chronic Obstructive Pulmonary Disease	4	2.72%
Hypothyroidism	4	2.72%
Prostate Adenoma	4	2.72%
Obesity	4	2.72%
Hepatitis B	3	2.04%
Sleep Apnea	3	2.04%
Tuberculosis	3	2.04%
Gastritis	3	2.04%
Epilepsy	3	2.04%
Congestive Heart Failure	2	1.36%
Pulmonary Embolism	2	1.36%
Non-Hodgkin Lymphoma	2	1.36%
Others *	11	7.48%

* Other comorbidities among the study cohort with an incidence of 1 patient (0.68%) each: gastrointestinal bleeding, deep vein thrombosis, aorta aneurysm, lung cancer, mitral valve disease, thyroid cancer, nephrolithiasis, melanoma, dementia, pituitary adenoma, colon cancer.

## Data Availability

The authors confirm that the data supporting the findings of this study are available within the article.
